# Vasomotor Regulation of Coronary Microcirculation by Oxidative Stress: Role of Arginase

**DOI:** 10.3389/fimmu.2013.00237

**Published:** 2013-08-19

**Authors:** Lih Kuo, Travis W. Hein

**Affiliations:** ^1^Department of Medical Physiology, Scott & White Healthcare, Texas A&M Health Science Center, Temple, TX, USA; ^2^Department of Surgery, College of Medicine, Scott & White Healthcare, Texas A&M Health Science Center, Temple, TX, USA

**Keywords:** endothelium, superoxide, nitric oxide, inflammation, arterioles, vasodilation

## Abstract

Overproduction of reactive oxygen species, i.e., oxidative stress, is associated with the activation of redox signaling pathways linking to inflammatory insults and cardiovascular diseases by impairing endothelial function and consequently blood flow dysregulation due to microvascular dysfunction. This review focuses on the regulation of vasomotor function in the coronary microcirculation by endothelial nitric oxide (NO) during oxidative stress and inflammation related to the activation of L-arginine consuming enzyme arginase. Superoxide produced in the vascular wall compromises vasomotor function by not only scavenging endothelium-derived NO but also inhibiting prostacyclin synthesis due to formation of peroxynitrite. The upregulation of arginase contributes to the deficiency of endothelial NO and microvascular dysfunction in various vascular diseases by initiating or following oxidative stress and inflammation. Hydrogen peroxide, a diffusible and stable oxidizing agent, exerts vasodilator function and plays important roles in the physiological regulation of coronary blood flow. In occlusive coronary ischemia, the release of hydrogen peroxide from the microvasculature helps to restore vasomotor function of coronary collateral microvessels with exercise training. However, excessive production and prolonged exposure of microvessels to hydrogen peroxide impairs NO-mediated endothelial function by reducing L-arginine availability through hydroxyl radical-dependent upregulation of arginase. The redox signaling can be a double-edged sword in the microcirculation, which helps tissue survival in one way by improving vasomotor regulation and elicits oxidative stress and tissue injury in the other way by causing vascular dysfunction. The impact of vascular arginase on the development of vasomotor dysfunction associated with angiotensin II receptor activation, hypertension, ischemia-reperfusion, hypercholesterolemia, and inflammatory insults is discussed.

A normal function of the vascular endothelium involving responses to physical (1), chemical (2, 3), and electrical (4, 5) stimuli is essential to maintain microvascular homeostasis and regulate local blood flow by changing vasomotor activity via release of endothelium-derived vasodilators, e.g., nitric oxide (NO), prostacyclin (PGI_2_), C-type natriuretic peptide, and hyperpolarizing factors (EDHF). The endothelium also releases vasoconstrictors such as endothelin-1, prostaglandin H/F, thromboxane, and angiotensin. Endothelial dysfunction is one of the earliest markers of vascular abnormalities observed in many cardiovascular diseases associated with oxidative stress due to excessive production of reactive oxygen species (ROS). Redox regulation of proteins by moderate levels of ROS is indispensable for signaling pathways underlying the regulation of subcellular and cellular activity as well as cardiovascular function (6–8). Notably, superoxide and hydrogen peroxide (H_2_O_2_) are the most common and important ROS involved in the physiological and pathophysiological events (6–8).

Superoxide is produced by several enzyme systems in the cell and it is converted to H_2_O_2_ by superoxide dismutase. H_2_O_2_ itself is a potent oxidizing agent that can be converted to hydroxyl radical in the presence of ferric compounds. H_2_O_2_ can be degraded by catalase to form H_2_O and an oxygen molecule. Compared with superoxide, H_2_O_2_ is stable, lacks charge, has longer half-life, is cell permeable, and can diffuse across longer distances. Therefore, its physical properties are suitable for second-messenger signaling (7, 8). Because a proper delivery of oxygen and nutrients to the tissue is essential for the normal function of an organ, in this review we will discuss the roles of superoxide and H_2_O_2_ in the physiological and pathophysiological regulation of vasomotor activity of resistance arterioles where blood flow is primarily controlled, with special focus on the coronary microcirculation. The deficiency of endothelium-derived vasodilators such as NO and PGI_2_ in relation to oxidative stress and the l-arginine consuming enzyme arginase is discussed.

## l-Arginine, Nitric Oxide Synthase, and Arginase

l-Arginine is the precursor for NO synthesis from three different isoforms of NO synthase (NOS). The endothelial NOS (eNOS) is the main isoform contributing directly to the regulation of vasomotor activity. In healthy human adults, it was estimated that 1.2% of arginine flux in the plasma contributes to the formation of NO and about 54% of whole body NO formation is derived from plasma arginine (9), although the fraction of l-arginine flux for NO production in the vasculature is unclear. Experimental data demonstrate that acute exogenous arginine provision can increase NO production (10, 11) and NO-mediated vasodilation (11, 12) despite the fact that the intracellular arginine level far exceeds the *K*_m_ of eNOS (13). It appears that the extracellular l-arginine exerts a significant impact on the synthesis of NO from the membrane-bound eNOS.

Besides NOS, arginase is another major l-arginine consuming enzyme, which converts l-arginine to ornithine and urea. Arginase is expressed most abundantly in the liver for ammonia detoxification via the urea cycle (14). Studies in the cardiovascular system have shown that endothelia (12, 15–18), vascular smooth muscle cells (12, 17, 19), macrophages (20, 21), and red blood cells (22), which do not possess the complete urea cycle enzymes, also express arginase. In humans, about 15% of plasma arginine flux is associated with extrahepatic arginase activity (9). There are two isoforms of arginase. Type 1 arginase (Arg-I) is cytosolic and predominately expressed in the liver. In extrahepatic tissues and cells, a low level of Arg-I expression has also been detected. Type 2, or mitochondrial, arginase (Arg-II) is expressed with low levels in brain, kidney, intestine, red blood cells, and immune cells. Arg-I and -II are derived from distinct genes located on different chromosomes (14) and can be induced or regulated independently by a wide array of agents/factors (23, 24). Although these two arginase isoforms are expressed in a variety of cells, their distribution varies with tissue/organ and cell types (25). In the vasculature, both isoforms of arginase have been identified and their expression is highly regulated for physiological and pathophysiological processes (17) but the relative level of expression may be species dependent (19, 26–28).

Synthesis and release of the vasodilator NO from eNOS, in response to various physiological or pharmacological stimulations, can be related to the substrate bioavailability (10, 11) and thus influence vasomotor activity (11, 12). In this regard, change of protein expression and activity of arginase is expected to have an impact on NO synthesis by affecting the l-arginine level. From the biochemical standpoint, the *K*_m_ of arginase for l-arginine in mammals, including humans, is reported to be around 0.5–29 mM (14). Although the *K*_m_ of NOS (1–20 μM) (29) is much lower than that of arginase, taking into consideration their *V*_max_ enzyme activities (1400 μmol⋅min^−1^⋅mg^−1^ for arginase vs. 900 μmol⋅min^−1^⋅mg^−1^ for NOS), the arginase is capable of competing with NOS for their substrate arginine (24). Based on the kinetic analysis of these two enzymes, the relative activity of NOS to arginase, in terms of consuming arginine, is diminished with either increasing arginine concentration or decreasing NOS to arginase molar ratio (24). Therefore, arginase activity can exceed NOS activity at higher levels of arginine or at higher arginase to NOS molar concentrations. Interestingly, the competition between NOS and arginase for arginine is more pronounced at lower levels of arginine (24). In terms of functional interpretation, the competitiveness (or importance) of arginase against NOS becomes apparent under conditions with upregulated arginase protein and limited supply of l-arginine.

Interestingly, intravenous administration of arginase causes constriction of cerebral arterioles and enhances platelet aggregation in mice (30), implicating that exogenous arginase may influence endothelial function through attenuation of NO production. However, the direct role of arginase in vasomotor regulation is unclear since the confounding effects from the changes in systemic hemodynamics and neuro-humoral factors cannot be excluded in this *in vivo* preparation. Using an isolated vessel approach, the role of endogenous arginase in vasomotor regulation of NO-mediated vasodilation was demonstrated for the first time in pressurized coronary arterioles (12). It was found that coronary arterioles express Arg-I in both endothelial and smooth muscle cells, and the NO production, as well as NO-mediated vasodilation, is enhanced by inhibiting arginase activity (12). It appears that endogenous arginase plays a counteracting role in the regulation of NO production and thus its associated vasomotor activity. The l-arginine-dependent NO-mediated vasodilation was also observed in various microvascular beds (11, 30–32) including human coronary arterioles (33), suggesting that l-arginine can be a limiting factor for the stimulated NO synthesis in the microcirculation. On the other hand, recent studies on cardiovascular diseases have implicated that upregulation of a specific arginase isoform in the vasculature may contribute to the development of vascular disease linked to l-arginine deficiency and reduced NO production (34, 35), especially under conditions with elevated level of angiotensin II (Ang II), hypertension, and inflammation, all of which are closely associated with oxidative stress (36).

### Vasomotor regulation by angiotensin II

In animal models of hypertension and myocardial hypertrophy, the excessive ROS release associated with renin-angiotensin system activation has been well documented (37, 38). However, the vasomotor action of Ang II in the intact heart is controversial. For example, a decrease (39, 40), an increase (41, 42), or a transient decrease followed by an increase (43, 44) in coronary blood flow by Ang II was reported. Although this inconsistency may be a result of using different animal models or experimental approaches, the complexity of flow regulation in the intact heart may be largely responsible for these diverse findings. Moreover, coronary vasomotor responses are influenced by the neural activity and by the changes in local hemodynamics and cardiac metabolism (44–46). The precise action of Ang II in the coronary microvasculature is difficult to assess in the intact heart because this peptide has direct and indirect actions on these biological factors (43, 44). It is also unclear whether the ROS generated by Ang II can modulate coronary microvascular reactivity in view that enhanced superoxide production by Ang II in endothelial cells is well recognized (47, 48).

Using isolated vessel approaches to eliminate the confounding influences from systemic and local effects inherited in *in vivo* preparations, it was found that Ang II, via activation of its type 1 (AT1) receptors, evokes a moderate vasoconstriction of porcine coronary arterioles (50–80 μm in diameter) at low concentrations (∼1 nM) but a marked vasodilation at higher concentrations (>10 nM) via AT2 receptor activation (49). This vasodilator effect is likely mediated by the released endothelial NO via bradykinin receptor signaling (50). Interestingly, in the human coronary circulation AT2 receptors were found expressed in the microvasculature only (50). Depending upon the concentration used, Ang II appears to exert different vasomotor activities in the coronary microvessels, and thus may explain the inconsistent observations on coronary flow changes *in vivo*. Moreover, pre-treating the isolated coronary arterioles with a sub-vasomotor concentration of Ang II (0.1 nM) for 60 min caused an elevation of superoxide production in the vessel wall and inhibited NO production and endothelium-dependent, NO-mediated dilation in response to adenosine, a potent metabolic vasodilator in the heart. This inhibitory effect was prevented by AT1 receptor blocker losartan, superoxide scavenger TEMPOL, or NAD(P)H oxidase inhibitor apocynin (49). These microvascular findings indicate that Ang II, at the level without causing vasomotor activity, exerts an adverse effect on NO-mediated vasodilator function via superoxide generated by AT1 receptor-dependent activation of NAD(P)H oxidase. Because acute myocardial ischemia (<60 min) upregulates the cardiac renin-angiotensin system and impairs coronary flow regulation (51, 52), it is speculated that the small elevation of local Ang II at sub-vasomotor levels in the heart during disease states may cause oxidative stress at the local microvascular domain and elicit focal vasoconstriction and myocardial ischemia secondary to the reduced NO bioavailability. In addition to the local vascular spasm, the deficiency of basal NO release from the endothelium, which is subjected to continuous shear stress stimulation, is expected to aggravate ischemic insult by promoting platelet aggregation and thrombosis formation (53, 54) in the microvasculature.

The blunted endothelium-dependent vasorelaxation in aging animals was recently reported to be associated with excessive vascular formation of ROS and upregulation of NAD(P)H oxidase subunits (e.g., Nox-1 and p22-phox), Arg-I, and AT1 and AT2 receptor expression in a manner sensitive to NAD(P)H oxidase inhibition and antioxidants (55). These findings suggest the initiation of vascular dysfunction by oxidative stress linking to Ang II receptors and arginase. However, the role and signaling pathway for Ang II receptor activation leading to NO deficiency in relation to arginase activity and vasomotor regulation is incompletely understood. In cultured bovine aortic endothelial cells, Ang II (0.1 μM, 24 h incubation) was recently shown to increase arginase activity and Arg-I expression through Gα_12/13_ protein-coupled AT1 receptor activation (56). The upregulated Arg-I appears to reduce l-arginine bioavailability and hamper NO production. The adverse effect of Arg-I is mediated by the activation of p38 mitogen-activated protein kinase (MAPK) pathways through RhoA/Rho kinase signaling (56). Although the threshold concentration of Ang II necessary for evoking NO deficiency and endothelial dysfunction in the above cell-culture study is unclear, chronic administration of Ang II (42 μg/kg/h, 2 weeks) in the mice was recently shown to impair endothelium-dependent NO-mediated relaxation of a tissue strip from corpus cavernosum (57). In agreement with the findings in cell culture (56), the Ang II-evoked endothelial dysfunction is mediated by the p38 MAPK-dependent upregulation of arginase (57). However, the responsible isozyme is Arg-II rather than Arg-I. Interestingly, inhibition of p38 MAPK not only prevents the effects of Ang II on endothelial function and arginase activity/expression, it also attenuates the increased systemic blood pressure by Ang II.

## Vasomotor Regulation in Hypertension

Hypertension is a major risk factor for coronary artery disease by impairing endothelium-dependent NO-mediated vasodilation (58) in the form of diminished bioavailability of NO, increased Ang II-dependent production of superoxide (59), and decreased endothelial levels of eNOS co-factor tetrahydrobiopterin (BH_4_) (60) or substrate l-arginine (61). In some studies, administration of l-arginine has been shown to restore endothelium-dependent vasodilator function in patients with essential hypertension (61) and to normalize coronary hemodynamics (62) and systemic blood pressure with enhanced NO production in hypertensive rats (63, 64). In deoxycorticosterone acetate (DOCA)-salt hypertensive rats, expression and activity of Arg-I protein in the aorta are elevated and correlate positively with blood pressure, suggesting the participation of this enzyme in pathophysiology of arterial hypertension (65). The upregulation of Arg-I in the coronary arteriolar wall was reported to contribute in part to the impairment of endothelial NO production and vasodilation by reducing l-arginine availability in hypertensive pigs (66). In the animal model of genetic (67, 68) or metabolic (69) form of hypertension, chronic inhibition of arginase was recently shown to improve endothelium-dependent vascular function (67–69), reduce cardiac fibrosis (68), prevent vascular remodeling and Arg-I overexpression (68), inhibit insulin-resistance (69), reduce oxidative stress (69), and alleviate hypertension (67–69). Although the evidence for the link of oxidative stress and inflammation to the pathogenesis of hypertension, and vice versa, is well supported in both experimental and clinical studies (70), it is unclear whether the direct elevation of mechanical stress on the vascular wall or the associated oxidative stress and inflammation contribute to the upregulation of vascular arginase during hypertension. Moreover, oxidative stress can probably promote inflammation and, conversely, inflammation *per se* may induce tissue damage and promote oxidative stress. Their individual contributions to the vasomotor dysfunction related to NO deficiency are difficult to define *in vivo* due to the complex and intertwined biological events and multifactorial processes involved in the development of vascular pathophysiology. However, recent studies using cell culture (71–75) and isolated vessel (49, 73, 76–80) approaches suggest that pro-inflammatory factors such as C-reactive protein (CRP), tumor necrosis factor-α (TNF-α), and oxidized low-density lipoprotein (Ox-LDL) are capable of causing endothelial NO deficiency and vasomotor dysfunction through elevated arginase and oxidative stress.

## Vasomotor Regulation by Pro-Inflammatory Factors

The dysfunction of coronary microvascular endothelial cells is closely associated with the development of various inflammatory diseases in the heart (81, 82). The inflammatory marker CRP, which has recently been established as a cardiovascular risk factor, also exhibits adverse effects on endothelium-dependent NO-mediated vasodilator function and NO production in isolated coronary (78) and retinal (83) arterioles by enhancing NAD(P)H oxidase-mediated superoxide production via p38 MAPK activation. Since activation of the endothelial p38 MAPK pathway by oxidative stress also has been documented (84, 85), the positive feedback between p38 MAPK and superoxide production is expected to exacerbate the oxidative insult on the vascular wall. In rats, treatment with human CRP at concentrations achievable in patients with cardiovascular disease impairs endothelium-dependent vasomotor function linked with uncoupling of eNOS due to reduction in dimerization of the enzyme, as well as inhibition of GTP cyclohydrolase I (GTPCH1), the rate-limiting enzyme in BH_4_ biosynthesis, and decrease in BH_4_ levels (86). Human CRP also causes the activation of NAD(P)H oxidase resulting in eNOS uncoupling directly or via inhibition of GTPCH1 or oxidation of BH_4_ (86). These studies provide the first evidence for the adverse action of human CRP *in vivo* manifested by impairing eNOS-dependent vasodilation and uncoupling of eNOS. Thus, given the importance of CRP-induced pro-oxidative effects and resultant eNOS inhibition, CRP appears to be a key molecule to accentuate endothelial dysfunction and contribute to blood flow dysregulation.

The CRP also displays an adverse effect on arachidonic acid-prostanoid pathways in the endothelium (79). The formation of peroxynitrite from NO (basal release) and superoxide (CRP-stimulated release) in the endothelium appears to compromise PGI_2_ production, and thus PGI_2_-mediated vasodilation, by inhibiting PGI_2_ synthase activity through tyrosine nitration (79). Peroxynitrite also contributes to eNOS uncoupling by oxidizing the co-factor BH_4_ (87) and thus reduces NO production. Although there is no evidence at the present time to suggest a direct impact of CRP on vascular arginase expression or activity, the elevation of serum Arg-I has been shown to correlate positively with CRP in asthmatic patients (88). Interestingly, the imbalance in l-arginine metabolism via arginase and NOS has been considered as a unifying element of asthma pathophysiology (89). The upregulation of arginase in the vasculature is expected to compromise endothelial NO with enhanced oxidative stress promoting peroxynitrite formation and hypertension during inflammatory insults in a manner similar to the development of allergic asthma in chronic inflammatory airway diseases (90) with primary and secondary forms of pulmonary hypertension (91–93). The recent report on the close relation between asthma and metabolic syndrome (94), a major risk of cardiovascular disease with dysregulation of l-arginine metabolism (69), supports the emerging role of arginase in the general regulation of NO production and oxidative stress in inflammatory diseases.

Tumor necrosis factor-α is a pro-inflammatory cytokine and an important mediator of cardiovascular complications such as acute myocardial infarction, ischemia-reperfusion injury, atherosclerosis, chronic heart failure, and coronary arterial disease in association with diminished coronary blood flow. Treating the isolated coronary arterioles with a pathological concentration of TNF-α (1 ng/ml, 90 min) caused a significant reduction of NO release, enhanced superoxide production, and c-Jun N-terminal kinase (JNK) phosphorylation in arteriolar endothelial cells and impaired endothelium-dependent dilation to adenosine (77). TNF-α participates in the pathogenesis and progression of myocardial injury induced by ischemia-reperfusion (95). In the model of ischemia-reperfusion injury of porcine coronary arterioles, the upregulation of Arg-I, via *de novo* protein synthesis pathway, causes endothelial dysfunction and NO deficiency (96). Using genetic tools to manipulate TNF-α expression in the mouse, it was found that myocardial ischemia-reperfusion evokes superoxide-dependent endothelial dysfunction and NO deficiency via upregulation of Arg-I, in a manner correlating with TNF-α expression (97). In contrast with the insults elicited by Ang II and CRP, the TNF-α induced oxidative stress and endothelial dysfunction are associated with the activation of ceramide-induced activation of JNK and subsequent production of superoxide via xanthine oxidase (77, 98) rather than the signaling via p38 MAPK-activated NAD(P)H oxidase (49, 78). Recent clinical evidence has shown that arginase blockade improves endothelium-dependent NO-mediated vasodilation in patients with coronary artery disease (99) and increases NO-dependent microvascular perfusion in patients with heart failure (100). Interestingly, the systemic level of Arg-I correlates with the severity of heart failure (100) and Arg-I polymorphisms are associated with myocardial infarction and vascular remodeling (101). The elevated level of Arg-I appears to be a major risk and/or pathogenic factor in developing coronary ischemic disease and vascular pathophysiology.

Experimental studies have shown that the expression of arginase is elevated in a variety of vascular and immune cells with inflammation and oxidative stress (20, 21, 102–104), the conditions that are known to be associated with atherogenesis. Interestingly, l-arginine deficiency coupled to impaired NO-mediated vascular function has been reported in animals (105–108) and humans (109–112) with hypercholesterolemia or atherosclerosis, possibly due to upregulation of arginase in the disease state (34). Furthermore, transgenic mice with overexpression of endothelial Arg-II exhibit increased aortic atherosclerotic lesions (113). In apolipoprotein E deficient mice, the arginase activity of atherosclerotic aorta is significantly elevated (28, 108). In the same mouse model, inhibition of arginase activity or deletion of Arg-II gene alleviates oxidative stress in the endothelium, prevents NO deficiency, and restores endothelial function, suggesting the critical role of Arg-II in triggering ROS-dependent endothelial dysfunction in hypercholesterolemia (114). Since Arg-II blockade reduces superoxide formation via a pathway sensitive to NOS inhibition (114), the uncoupling of eNOS, due to l-arginine deficiency, appears to be involved in the arginase-dependent oxidative stress. It was found that Arg-II activity positively correlates with RhoA protein level in atherosclerotic aortas and that manipulation of RhoA/Rho kinase activity and expression directly affects enzymatic activity of Arg-II (28). In this regard, RhoA/Rho kinase activation is likely responsible for the increased Arg-II activity leading to vascular dysfunction and atheroma formation. Rho kinase activation also contributes to Arg-I-mediated coronary vascular dysfunction in diabetic rats and to NO deficiency induced by hyperglycemia in bovine aortic endothelial cells (115). In the rabbit model of hypercholesterolemia, the expression of both arginase isozymes is elevated in atherosclerotic aortas (27). However, the regulation and role of specific arginase isoforms in disease development remains to be determined.

In the coronary microcirculation, the endothelium-dependent NO-mediated dilation, compared to that mediated by the EDHF and the endothelial prostanoids, is more susceptible to the insult of Ox-LDL (116) than that of native LDL (3). The enhanced superoxide production and reduced l-arginine bioavailability are responsible for the observed endothelial dysfunction of coronary arterioles (3). In cultured human aortic endothelial cells, Ox-LDL activates lectin-like Ox-LDL receptor-1 (LOX-1) and subsequently increases Arg-II activity/expression and reciprocally inhibits NO production via RhoA/Rho kinase activation (117). Interestingly, the NO deficiency, as well as the increased arginase activity and ROS production, evoked by hypercholesterolemia or Ox-LDL are not observed in endothelial cells absent of LOX-1, suggesting the critical role of LOX-1 in mediating arginase-dependent NO deficiency and oxidative stress (117). The accumulation of superoxide is likely derived from the uncoupled eNOS and NAD(P)H oxidase because blockade of these enzymes attenuates oxidative stress (117). In the intact porcine coronary arterioles, the upregulated Arg-I contributes, in part, to the reduced NO production and impaired endothelium-dependent dilation evoked by Ox-LDL (118). However, it is unclear whether LOX-1 plays a role in this experimental model.

## Vasomotor Regulation by H_2_O_2_

The elevated level of H_2_O_2_ has been detected under various pathophysiological conditions, including ischemia-reperfusion, inflammation, hypertension, diabetes, and atherosclerosis. The H_2_O_2_ can be released from various types of cells, including vascular cells (119, 120) and has been implicated, in some tissues, as an endothelium-derived hyperpolarizing factor exhibiting vasodilator activity (119). Extraluminal administration of H_2_O_2_ (1–100 μM) elicits concentration-dependent dilation of isolated coronary arterioles in part via an endothelium-dependent mechanism through cyclooxygenase (COX)-1-mediated release of PGE_2_ (121). H_2_O_2_ can also cause smooth muscle hyperpolarization and lead to vasodilation through the opening of calcium-activated potassium channels (121, 122). This vasodilator response plays a role in regulating coronary perfusion by recruiting blood flow to the heart during pressure reduction (i.e., autoregulation) (123) or metabolic activation (i.e., functional hyperemia) (124). Interestingly, in disease states, the vasodilator action of H_2_O_2_ appears to compensate for the impaired NO-mediated dilation linking to the uncoupling of eNOS with its co-factor BH_4_ (125) and to protect ischemia-reperfusion injury in the coronary microcirculation (126). In the pig model of coronary ischemia, the impaired NO-mediated vasodilation in collateral-dependent arterioles distal to chronic coronary occlusion was restored by exercise training (127). The beneficial effect of exercise on coronary arteriolar function was abolished by catalase, suggesting the contribution of H_2_O_2_ in compensating and restoring endothelium-dependent vasomotor function in the phase of collateral microvessel adaption to myocardial ischemia (127).

On the other hand, H_2_O_2_ can exert an adverse effect by reducing endothelial release of NO for vasodilation when the endothelium is exposed to a prolonged (e.g., 60 min) elevation of excessive H_2_O_2_ (e.g., 100 μM) (128). Interestingly, the dilation mechanisms involving the activation of COX, guanylyl cyclase, cytochrome-P450 monooxygenase, and potassium channels are not affected by H_2_O_2_ (128). Moreover, supplementation of l-arginine or inhibition of arginase restores H_2_O_2_-impaired vasomotor function, and the adverse effect of H_2_O_2_ can be prevented by inhibiting hydroxyl radical production (128). It appears that a high intravascular level of H_2_O_2_ selectively impairs endothelium-dependent NO-mediated dilation of coronary microvessels by reducing l-arginine availability. The formation of hydroxyl radicals leading to Arg-I overexpression is responsible for the adverse effect of H_2_O_2_ (128). Interestingly, it was recently shown that the oxidative stress elicited by peroxynitrite or H_2_O_2_ increases Arg-I activity/expression through protein kinase C-mediated activation of RhoA/Rho kinase in bovine aortic endothelial cells (129). It remains unclear whether hydroxyl radicals and protein kinase C contribute to the activation of Rho kinase in intact microvessels.

## COX and Arginase in Vascular Regulation

Although COX activation is known to mediate tissue inflammation and participate in vasomotor regulation (130), its linkage to arginase, another important enzyme related to the inflammation process (89), remains unclear. A recent study has shown that inhibition of arginase improves endothelial function and attenuates vascular COX-2, thromboxane synthase, and PGI_2_ synthase activities in the rat model of adjuvant-induced arthritis (131). Thus, arginase activation contributes to the augmentation of inflammatory enzyme activity related to prostanoid synthesis. Interestingly, arginase inhibition improved endothelial function, but it had no effect on the arthritis severity of the animal (131). It appears that this type of inflammatory insult targets vascular arginase and consequently leads to vascular disorder. While COX-2 inhibitors have been shown to reduce tumor growth through arginase inhibition (132, 133), administration of diclofenac, a non-steroidal anti-inflammatory drug against COX-2 (134) and phospholipase A_2_ (135), was found to cause tumor suppression via a mechanism related to the inhibition of tumor vascularization (136). Although the expression and activity of arginase in the vasculature was not evaluated in this study, it is speculated that the observed tumor suppression is attributable to the inhibition of vascular arginase since this enzyme has been shown to play an important role in the growth of vascular cells (35, 137–139). Although the direct link between COX and arginase in vasomotor regulation remains to be determined, the finding of the close association between these two enzymes in tumor-promoted angiogenesis (140) and in alleviating chronic hypertension and improving vascular endothelial function and vasomotor activity (68) may provide new direction and insights into this underdeveloped research area.

## Arginase Isozymes and Vasomotor Dysfunction

The arginase inhibitors currently available are not isoform selective and their specificity may be species dependent (17). Therefore, it is difficult to identify the role and function of a specific arginase isoform using pharmacological tools. With above limitations, genetic manipulation of an arginase isoform becomes an important strategy for more precise study of arginase function in a living system. Homozygous deletion of Arg-I is lethal to the animal in the perinatal period (141). In contrast, homozygous deletion of Arg-II in the mice does not cause significant changes in phenotype, except an elevation of plasma level of arginine (142). The observed increase in endothelial NO production and NO-mediated vasorelaxation, in conjunction with reduced vasoconstrictor response, in carotid arteries from Arg-II knockout mice (143) supports the idea that endothelial Arg-II plays a counteracting role in NO production and the associated vasomotor dysfunction. Deletion of Arg-II gene attenuates vascular disorder (i.e., impaired NO-mediated endothelial function and enhanced sympathetic vasoconstriction) in corpora cavernosal tissue of mice with type 1 diabetes, suggesting the detrimental role of Arg-II in this disease model (144). Arg-II appears to modulate not only vasomotor reactivity but also the physical property of the vascular wall by influencing NOS activity because Arg-II deficient mice exhibit decreased vascular stiffness in a manner sensitive to NOS inhibition (143). On the other hand, selective overexpression of human Arg-II gene in the endothelium causes systemic hypertension, impairs endothelium-dependent NO-mediated vasorelaxation, and promotes atherosclerotic lesions (113). These *in vivo* findings are in agreement with the observed adverse effect of Arg-II on NO-mediated endothelial function in cell culture. Moreover, the experimental data from an Arg-II knockout study indicate that the renal injuries observed in spontaneous or streptozotocin-induced diabetes animals are also mediated by Arg-II (145). However, genetic manipulation of Arg-I (partial deletion) in Arg-II deficient mice shows that upregulation of vascular Arg-I, rather than Arg-II, contributes to the diabetes (type I)-induced endothelial dysfunction, vascular stiffness, and coronary fibrosis (146), in which Rho kinase activation can be responsible for the observed pathophysiology (147). Using the same genetic approach, the detrimental role of Arg-I in mediating blood pressure elevation and vascular endothelial dysfunction was recently reported in the mice subjected to systemic hypertension induced by DOCA-salt (148). In diabetic human patients, Arg-I upregulation appears to be responsible for the impairment of coronary arteriolar dilation to an endothelium-dependent NO-mediated agonist (149).

Surprisingly, a recent study by Huynh et al. showed that Arg-II knockout mice start to display hypertension at 8 weeks old, despite the reduction in vasoconstrictor responsiveness (150). The observed changes in systemic hemodynamics are associated with left ventricular hypertrophy, diastolic dysfunction, and increased sympathetic activity (150). In contrast to the previous report in carotid arteries with Arg-II deletion by Lim et al. (143), the aortic relaxation to an NO-dependent agonist was not significantly enhanced in the Arg-II knockout mice (150), suggesting that the observed reduction in the vasoconstrictor response was not attributable to alterations in NO production. There is no clear explanation to the apparent discrepancies between these two Arg-II knockout studies, especially in the observed global changes in cardiovascular function and vasomotor regulation related to endothelial NO. Nevertheless, the study of Huynh et al. demonstrated a correlation between Arg-II and Rho kinase, suggesting a contribution of downregulation of Rho kinase to the observed reduction in the vasoconstrictor response in Arg-II deficiency (150). This is in agreement with the context that upregulation of arginase in the disease state may enhance Rho kinase activity/expression and consequently alter vasomotor activity because numerous studies have implicated a close association between Rho kinase and arginase in the development of vascular dysfunction (28, 56, 115, 117, 129).

Although recent studies using genetic approaches have provided significant insights into the contribution of specific arginase isozymes in vasomotor regulation in health and disease, the inconsistent results are often reported as discussed above. In view that arginase gene deletion might also alter expression of other genes or activate alternate signaling pathways to confound the consequences of initial gene deletion (151, 152), the interpretation of these results should be cautious. The gene–gene interaction and the development of compensatory and/or decompensatory biological responses, at local or systemic levels, with gene manipulation may contribute to the observed discrepancies, in addition to the variation of involved signaling molecules, age, gender, tissue/organ, species/strain, and experimental conditions.

At the protein level, although Arg-I and -II carry out the same catalytic function, they have different physicochemical characteristics, including immunological cross-reactivity, charge, and subcellular location (14). Because the expression pattern of specific arginase isoforms can be cell/tissue and animal species dependent (17), it is unclear at the present time why and how Arg-I and -II can be targeted differently. Interestingly, recent studies suggest that the catalytic efficiency of arginase can be modulated without altering protein expression (153). It appears that cysteine residues 303 in Arg-I can undergo S-nitrosylation and subsequently increase stability of the arginase trimer and reduce its *K*_m_ for arginine (153). This increase in arginase activity can contribute to the endothelial dysfunction and reduced NO bioavailability (153). On the other hand, there is no cysteine in mammalian Arg-II that corresponds to cysteine 303 in Arg-I, suggesting that the post-translational modulation via S-nitrosylation might not occur in Arg-II (17). It is likely that S-nitrosylation elicited by the excessive production of NO during iNOS induction (e.g., inflammation) or by the formation of peroxynitrite during oxidative stress may contribute to a selective activation of Arg-I leading to endothelium-dependent vasomotor dysfunction. Moreover, uric acid has been demonstrated to increase arginase activity by increasing the affinity for arginine (154). This phenomenon is unlikely isoform selective because it is observed in the pulmonary arterial endothelial cell lysates (Arg-II) and rat kidney (Arg-II) and liver (Arg-I) homogenates (154). However, it was recently found that uric acid, at the concentrations reported to affect arginase activity (154), does not alter Arg-II activity in cultured human umbilical vein endothelial cells (155). The explanation for these inconsistent findings on uric acid-arginase interaction remains unclear. Hydroxyl radicals derived from H_2_O_2_ appear to specifically induce Arg-I expression and lead to endothelial dysfunction in coronary microvessels (128). Interestingly, biochemical studies *in vitro* indicate that Arg-I enzyme activity can be enhanced by hydroxyl radicals (156). Although it has not been demonstrated whether hydroxyl radicals also alter Arg-II activity, the activation of Arg-I, both in protein expression and activity, by oxidative stress (i.e., peroxynitrite and H_2_O_2_) in cultured endothelial cells also has been reported recently (129). In view that the increase of Arg-I activity (50%) is more than that of protein expression (35%) (129), the direct impact of these insults on arginase enzyme activity *per se* is apparent. Collectively, the above studies suggest the differential activation of arginase isozymes, depending upon the environment and the nature of the stimulation, in addition to the selective regulation of its protein expression in the vasculature. These differential regulation mechanisms may also contribute to the observed diversity and heterogeneity in involved arginase isoforms in vascular cell, as well as the exerted function, in different tissues, species, and diseases. Further studies on the differential activation of specific arginase isoforms are required.

## Conclusion and Perspectives

**Figure 1:**
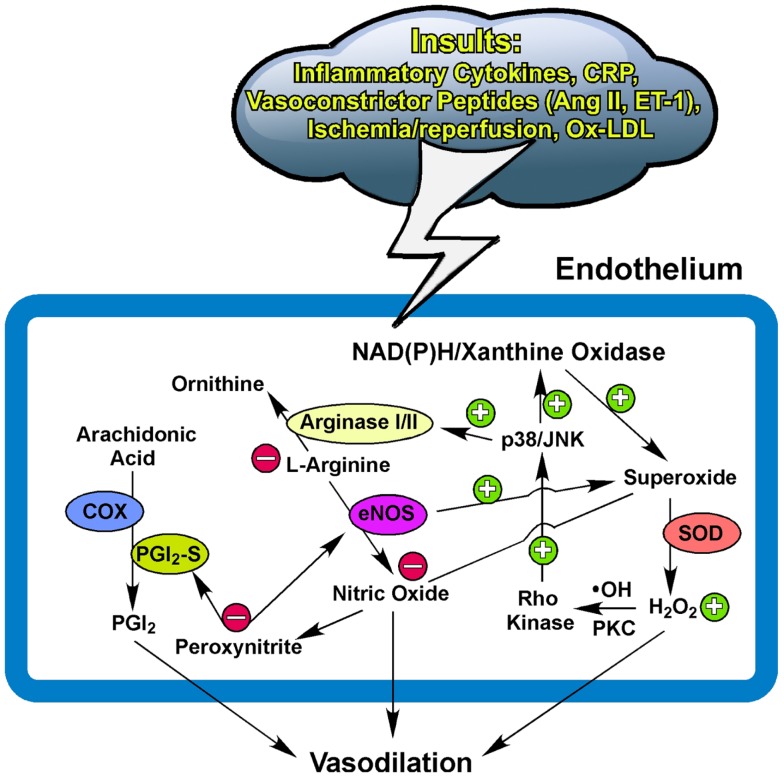
**Potential pathways for redox and arginase modulation of vasomotor function**. The low level of superoxide and hydrogen peroxide (H_2_O_2_) is essential for maintaining normal homeostasis of the endothelium to exert vasodilation in response to physiological stimulation. The excessive production of superoxide from the activated NAD(P)H oxidase and/or xanthine oxidase by insults from inflammatory cytokines, C-reactive protein (CRP), ischemia/reperfusion, oxidized low-density lipoprotein (Ox-LDL), or vasoconstrictor peptides such as angiotensin II (Ang II) and endothelin-1 (ET-1) (162–165) scavenges the released nitric oxide and subsequently forms peroxynitrite. The prolonged and elevated production of H_2_O_2_ from superoxide dismutase (SOD) suppresses NO production by up-regulating vascular arginase via p38 mitogen-activated protein kinase or c-Jun-N-terminal kinase (JNK) signaling following the hydroxyl radical (∙OH)- or protein kinase C (PKC)-mediated activation of Rho kinase. The upregulated arginase limits substrate L-arginine availability to nitric oxide synthase (eNOS) for nitric oxide synthesis and also uncouples eNOS to promote superoxide production. The production of nitric oxide from eNOS and prostacyclin (PGI_2_) from cyclooxygenase (COX) and PGI_2_ synthase (PGI_2_-S) is inhibited by peroxynitrite due to uncoupling of eNOS and nitration of PGI_2_-S (see text for details). Peroxynitrite also activates arginase and consequently reduces nitric oxide production. These redox events, in company with arginase upregulation, compromise endothelial function and thus augment vascular tone and reduce nitric oxide-mediated arteriolar dilation for blood flow recruitment and regulation. +, enhance/increase/upregulate; −, inhibit/reduce/downregulate.

Collectively, the NO bioavailability, determined by the synthesis/release and utilization/scavenging at the level of the endothelium, plays an important role in maintaining vascular homeostasis and function, as well as disease development linking to oxidative stress and inflammation. Redox signaling with a low level of ROS released from cardiomyocytes and/or vascular cells displays an indispensable role in maintaining microcirculatory homeostasis by regulating vasomotor activity in response to physiological challenges. The release of H_2_O_2_ from the vasculature helps to restore vasomotor function by compensating for NO deficiency in coronary collateral microvessels adapted to chronic myocardial ischemia with exercise training. Depending upon the disease model and the pathophysiological insult, the excessive and prolonged production of superoxide, via stress kinase-activated NAD(P)H oxidase or xanthine oxidase, and the subsequent exorbitant formation of H_2_O_2_, appear to generate oxidative stress and inflammation, which outweighs the benefits of vasoregulation by impairing endothelial function and possibly exhausting vasodilator reserve (Figure 1). The status and the balance of redox signaling in the vascular cells and their surrounding parenchymal tissues appear to modulate the vasomotor function of microvessels in health and disease.

The converging evidence suggests that NO-mediated vascular function, including vasomotor activity, can be influenced by the arginase activity in the endothelium and/or its surroundings. The upregulation of arginase, in either protein or activity, contributes to vascular dysfunction in various vascular diseases by initiating or following oxidative stress and inflammation (Figure 1). Therefore, therapeutic inhibition of arginase may be useful for disease treatment. However, a global Arg-II deletion can develop hypertension, ventricular hypertrophy, and cardiac dysfunction with age (150). Because these cardiovascular disorders are not present at young age with Arg-II ablation, chronic Arg-II deficiency appears to elicit a series of cardiovascular remodeling (e.g., compensation and decompensation). Moreover, biochemical studies indicate that Arg-I and -II can exhibit different enzyme kinetics for substrate binding and products, as well as different sensitivities and responsiveness toward inhibitors (17, 157). These isozyme-dependent characteristics, in combination with the use of different experimental models and animal species, may complicate the experimental results, interpretations, and conclusions on the effect of arginase inhibition on endothelial function and vasomotor regulation under physiological and pathophysiological conditions. In this regard, the clinical benefits of inhibition of specific arginase isoforms for cardiovascular disease treatment are uncertain and deserve further investigation. It is worth noting that the systemic supplementation of antioxidants showed no benefit but instead promoted possible harmful effects in cardiovascular disease prevention or therapy (158–161). Oxidative stress and inflammation are two sides of the same coin and can be the cause or result of arginase upregulation in the vasculature via diverse signaling mechanisms. Localized manipulation of the redox system and arginase activity in a diseased vessel might be a useful strategy to improve flow regulation and thus enhance oxygen and nutrient delivery for tissue survival and recovery.

## Conflict of Interest Statement

The authors declare that the research was conducted in the absence of any commercial or financial relationships that could be construed as a potential conflict of interest.
